# High Genetic Diversity and Novelty in Eukaryotic Plankton Assemblages Inhabiting Saline Lakes in the Qaidam Basin

**DOI:** 10.1371/journal.pone.0112812

**Published:** 2014-11-17

**Authors:** Jiali Wang, Fang Wang, Limin Chu, Hao Wang, Zhiping Zhong, Zhipei Liu, Jianyong Gao, Hairong Duan

**Affiliations:** 1 State Key Laboratory of Simulation and Regulation of Water Cycle in River Basin, China Institute of Water Resources and Hydropower Research, Beijing, China; 2 Institute of Shandong River Wetlands, Laiwu, China; 3 College of Hydrology and Water Resource, Hohai University, Nanjing, China; 4 Institute of Microbiology, Chinese Academy of Sciences, Beijing, China; 5 Genewiz, Inc, Beijing, China; Missouri University of Science and Technology, United States of America

## Abstract

Saline lakes are intriguing ecosystems harboring extremely productive microbial communities in spite of their extreme environmental conditions. We performed a comprehensive analysis of the genetic diversity (18S rRNA gene) of the planktonic microbial eukaryotes (nano- and picoeukaryotes) in six different inland saline lakes located in the Qaidam Basin. The novelty level are high, with about 11.23% of the whole dataset showing <90% identity to any previously reported sequence in GenBank. At least 4 operational taxonomic units (OTUs) in mesosaline lakes, while up to eighteen OTUs in hypersaline lakes show very low CCM and CEM scores, indicating that these sequences are highly distantly related to any existing sequence. Most of the 18S rRNA gene sequence reads obtained in investigated mesosaline lakes is closely related to Holozoa group (48.13%), whereas Stramenopiles (26.65%) and Alveolates (10.84%) are the next most common groups. Hypersaline lakes in the Qaidam Basin are also dominated by Holozoa group, accounting for 26.65% of the total number of sequence reads. Notably, Chlorophyta group are only found in high abundance in Lake Gasikule (28.00%), whereas less represented in other hypersaline lakes such as Gahai (0.50%) and Xiaochaidan (1.15%). Further analysis show that the compositions of planktonic eukaryotic assemblages are also most variable between different sampling sites in the same lake. Out of the parameters, four show significant correlation to this CCA: altitude, calcium, sodium and potassium concentrations. Overall, this study shows important gaps in the current knowledge about planktonic microbial eukaryotes inhabiting Qaidam Basin (hyper) saline water bodies. The identified diversity and novelty patterns among eukaryotic plankton assemblages in saline lake are of great importance for understanding and interpreting their ecology and evolution.

## Introduction

Saline lakes usually occur in endorheic drainage basins, which span approximately 1/10 of the Earth's surface area [1]. Inland saline lakes represent approximately 5% of modern drylands [2]; these lakes are numerous and are distributed worldwide in semi-arid or arid areas [3]. Inland saline lakes and freshwater lakes from humid areas account for similar proportions of global water, approximately 0.008% and 0.009%, respectively [4]–[5]. Saline lakes are important reservoirs of largely unseen microbial biodiversity with high phylogenetic richness and novelty [5]. Saline lakes at high altitudes are also productive and represent an important and extreme ecosystem harboring many novel prokaryotic microorganisms [6]–[7]. Small-sized planktonic microorganisms are critical for aquatic systems, mostly as major contributors to production and biomass and as key players driving carbon and nutrient cycles [8]–[9]. The genetic diversity of microbial communities in saline lakes has been studied in different areas of the world, including the USA [10]–[11], Mongolia [12], China [7], Iran [13], Australia [14], Spain [5], [15], and the Andean Altiplano [16]. However, our current knowledge on microorganisms isolated in culture does not completely represent the microbial diversity in saline systems [5], [7], [15], [17].

Salinity is an important factor that selects and structures microbial assemblages globally [18]–[20], and microorganisms inhabiting high salinity environments, mostly prokaryotes, have developed several salinity-stress adaptation strategies [21]. Eukaryotes might have greater difficulty in coping with the selective effect of high salinity [21]–[22], resulting in large decreases in the number of species as salinity increases [23]. This hypothesis might explain why eukaryotes are poorly represented in high-salinity environments compared to prokaryotes. Description of the molecular diversity of small marine eukaryotes through rRNA gene cloning and sequencing has revealed a large diversity of ribosomal types and identified novel lineages within microbial eukaryotes [24]–[25]. However, there are few studies analyzing the genetic diversity of eukaryotic assemblages in high-salt environments at high altitudes, although consistent changes in eukaryotic community composition and richness have been observed along salinity gradients [26]. Sequence analysis of selected major denaturing gradient gel electrophoresis (DGGE) bands revealed many sequences (largely protist) that are not related to any known cultures but that are related to uncultured eukaryotic picoplankton and unidentified eukaryotes in Eastern Tibetan Lakes [7]. High-salinity water bodies in inland saline ponds contain an unexpected large genetic diversity of novel protists [15], but the number of such eukaryotic microbial species in these environments remains to be elucidated [5], [27].

Traditionally, studies on the diversity of eukaryotic assemblages (protist) have largely relied on morphological surveys using different microscopic techniques [28]–[30], and some important components of the microbial diversity in environmental samples have remained undetected using traditional methods [5], [15]. Microscopy approaches have difficulties in identifying small cells (<10 µm), and thus, this fraction is understudied [25]. Recently, the development of high-throughput next-generation sequencing (NGS) technology for DNA sequencing [5], [27], [30]–[33] has facilitated extensive sequence-based characterization of diverse natural microbial communities and has allowed an assessment of microbial communities at high resolution based on deep taxon sampling [34]. Because millions of sequence reads are generated in a single experiment, NGS has revolutionized surveys of microbial diversity. Compared to microscopy, NGS-based amplicon sequencing is superior in detecting rare species [35], and it is now possible to recognize and identify nano- and picophytoplankton such as unicellular cyanobacteria and small flagellates, which cannot be discriminated based on morphological features [25], [27], [30], [33].

The 18S rRNA gene is a widely used and valuable ‘bar-code’ to analyze eukaryotic diversity, because it is universally present in living organisms, and there are significant sequence data for comparison in public databases such as GenBank [30], [33], [36]. Recently, this gene marker is commonly used for the next-generation sequencing [25], [41]–[42]. The repetitive arrangement of rRNA genes within the genome provides large amounts of template DNA for PCR, even in the smallest organisms. Comparative studies using 18S rRNAs have observed that the major length-variable regions are distributed on the surface of the molecules [43]–[45], whereas the intronic splicing sites are clustered in the inner region [45]. However, some sources of bias have been identified, including pyrosequencing errors [46–47], inappropriate clustering approaches [48] or inconsistent results from different targeted 18S rDNA regions [42]. More recently, Hadziavdic [33] et al. (2014) completely characterized the variable and conserved regions in the 18S rRNA gene, and their results suggested that the V2, V4, and V9 regions are best suited for biodiversity analysis. Furthermore, algorithms to remove pyrosequencing errors have been developed to reduce these overestimations, such as single-linkage preclustering (SLP) approach [48]. The 18S rDNA amplicon pyrosequencing has become a widespread approach for microbial community diversity studies. However, to the best of our knowledge, no similar studies have been performed on eukaryotic plankton in high-salt environments at high altitudes.

The Qaidam Basin is located in the northeastern section of the Plateau of Tibet, occupying the northwestern part of Qinghai province, western China. The basin is almost entirely an area of interior drainage, with rivers discharging either into Koko Nor or into one of the numerous salt lakes and saline swamps in the basin’s central area, such as Tuosu, Dasugan, Gahai, Gasikule and Xiaochaidan. In the present work, we have analyzed the genetic diversity of planktonic microbial eukaryotes (size range 0.2–20 µm) along a salinity gradient in six water bodies in the Qaidam Basin using next generation sequencing (NGS). Samples were obtained from different geographic regions and covered a wide range of environmental conditions such as salinity (concentration and composition), in situ temperatures, trophic status, water and connectivity regimes, and altitude, which captured part of the high variety of saline habitats present in continental areas.

## Materials and Methods

### Sample collection and DNA extraction

**Figure 1:**
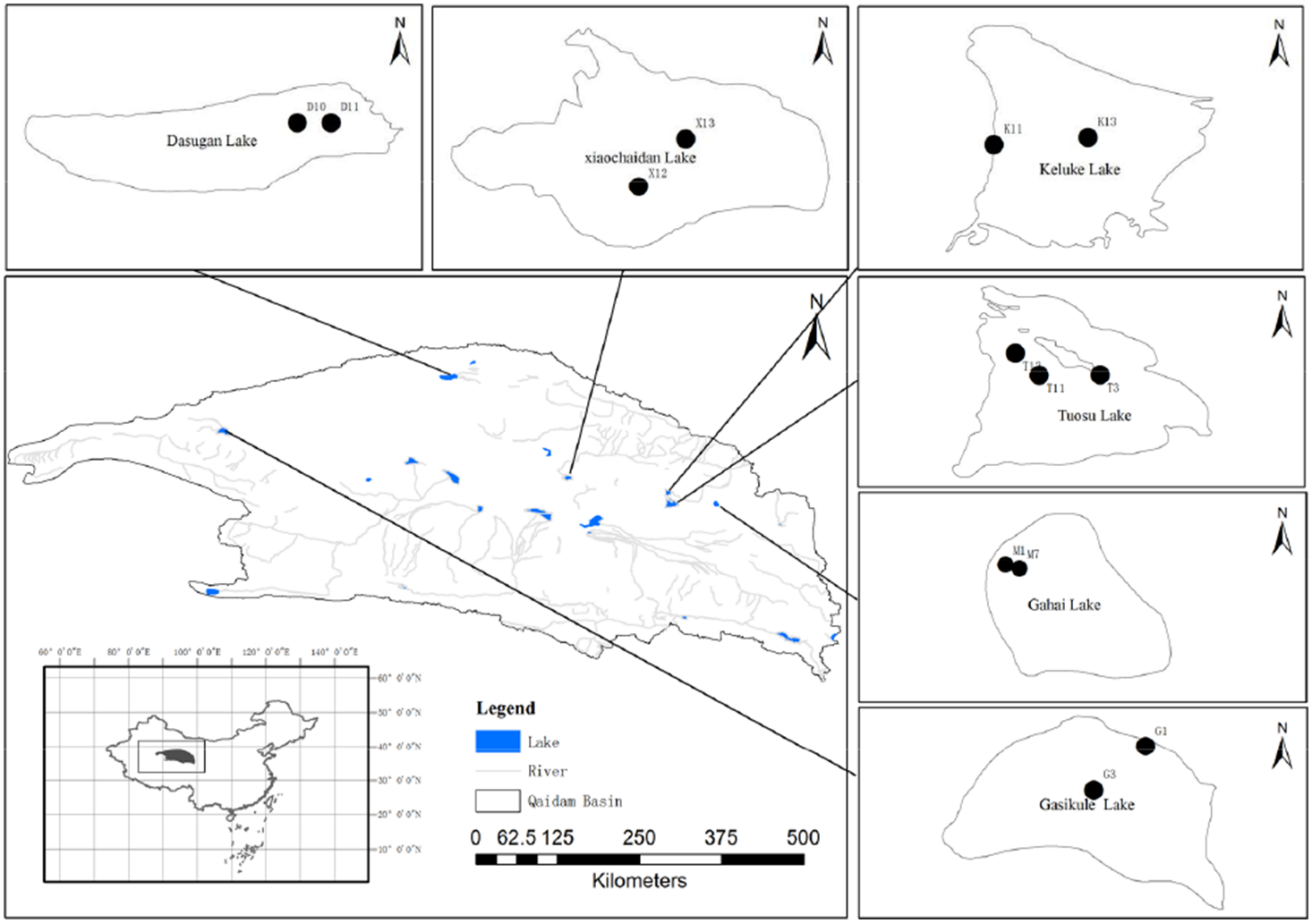
Locations of investigated lakes in Qaidam Basin. In Figure, K, T, D, M, X and G referred to Lake Keluke, Lake Tuosu, Lake Dasugan, Lake Gahai, Lake Xiaochaidan and Lake Gsikule, respectively.

No specific permissions are required for six lakes, located in publicly accessible areas. The field studies did not involve endangered or protected species. Overall, 13 samples (Lake Keluke 1, Lake Keluke 2, Lake Dasugan 10, Lake Dasugan 11, Lake Tuosu 3, Lake Tuosu 11, Lake Tuosu 12, Xiaochaidan 12, Xiaochaidan 13, Lake Gahai 1, Lake Gahai 7, Lake Gsikule 1 and Lake Gsikule 3, hereafter referred to as K11, K13, D10, D11, T3, T11, T12, M1, M7, X12, X13, G1, G3, respectively) were analyzed (Table 1). Six lakes located on the Qaidam Basin at altitudes ranging from 2,792 to 3,170 m above sea level were investigated (Figure 1). The lakes were chosen in order to cover a salinity gradient from 0 to 466 g/L. Water samples were collected from surface waters (top 30 cm) with a 5 L schindler sampler in August 2013 and were first condensed by 0.2-µm pore-size filters. Plankton samples (2000–2500 ml water) for NGS analyses were further filtered through a 20 µm mesh sieve immediately to remove most of the large particles. Water temperatures, pH and dissolved oxygen levels were measured on board using a Hydrolab sensor (Austin, TX, USA). Concentrations of the six major ions potassium (K^+^), sodium (Na^+^), calcium (Ca^2+^), magnesium (Mg^2+^), chloride (Cl^−^), and sulfate (SO_4_^2−^), as well as the concentration of total nitrogen (TN) and total phosphorus (TP) were measured according to the standard methods [5], [7], [49] after transportation of samples to the laboratory. The total dissolved solid (TDS) of the investigated habitats was determined by a conventional conductivity meter (Table 1). Filters for extraction of DNA were centrifuged to the bottom of the eppendorf tube (3200 g 5 min, Eppendorf 5810R) and stored at −80°C in an ultra low-temperature freezer for DNA extraction. DNA was extracted after the cetyltrimethylammonium bromide extraction procedure [7]. The final ethanol-rinsed DNA pellets were dried and resuspended in 100 µL of 1× TE buffer (10 mM Tris–HCl, 1 mM EDTA) and stored at −80°C. DNA concentration and quality were determined with a NanoDrop 1000 spectrophotometer (Wilmington, DE, USA).

**Table 1:** The geographical, physical, and chemical characteristics of the investigated lakes. *ND* not determined, *n.d*. not detectable.

	SampleID	Longitude	Latitude	TNmg/L	NH_3_-Nmg/L	PH	D0 mg/L	Samplingdepth	Temperature°C	Altitude m	TDSg/L	Na^+^g/L	K^+^g/L	Mg^2+^g/L	Ca^2+^g/L	Cl^−^g/L	SO_4_^2−^g/L
**Fresh** **water lake**	Keluke11	96°51′39.05″	37°17′01.98″	2.21	0.40	8.83	8.7	0.3	15.1	2807	*ND*	*n.d*	*n.d*	*n.d*	*n.d*	*n.d*	*n.d*
	Keluke13	96°53′57.04″	37°17′12.23″	1.57	0.33	8.89	9.7	0.3	15.6	2816	*ND*	*n.d*	*n.d*	*n.d*	*n.d*	*n.d*	*n.d*
**Mesosaline** **lake**	Tuosu3	96°57′26.00″	37°08′59.00″	2.45	1.26	8.63	5.7	0.3	17.9	2803	30.9	7.47	0.22	1.5	0.04	12.36	6.52
	Tuosu11	96°54′54.27″	37°08′58.91″	2.74	0.36	8.82	6.5	0.3	17.1	2802	30.3	7.37	0.22	1.31	0.03	12.09	6.39
	Tuosu12	96°53′34.00″	37°19′58.00″	3.60	0.23	8.9	6.6	0.3	15.3	2801	30.5	*ND*	*ND*	1.25	0.04	10.42	6.37
	Dasugan10	93°56′02.39″	38°52′48.02″	0.55	0.40	8.95	6.4	0.3	13.2	2796	31.2	6.94	0.39	1.16	0.09	9.29	9.41
	Dasugan11	93°57′21.25″	38°52′48.02″	4.19	0.94	8.98	7	0.3	12.8	2792	30.7	6.57	0.37	1.08	0.11	9.16	9.28
**Hypersaline** **lake**	Gahai1	97°31′32.45″	37°08′41.78″	2.05	0.46	8.19	2.8	0.3	19.3	2853	96.9	24.5	0.39	1.06	0.27	45.65	11.63
	Gahai7	97°31′52.61″	37°08′36.19″	3.11	0.4	8.19	2.2	0.3	19.3	2851	93.6	25.02	0.41	1.23	0.27	45.02	11.52
	Xiaochadan12	95°30′19.03″	37°28′31.77″	3.27	1.08	8.31	4.9	0.3	10.8	3170	96.7	31.43	0.56	1.3	0.39	44.68	18.19
	Xiaochadan15	95°33′9.10″	37°28′52.57″	3.29	1.07	8.32	4.9	0.3	10.5	3168	96.2	*ND*	*ND*	1.71	0.41	42.76	4.08
	Gasikule1	90°49′01.89″	38°09′43.03″	8.9	1.18	6.73	1.4	0.3	16	2858	348.3	74.65	2.46	2.96	0.16	134.28	10.75
	Gasikule3	90°46′59.72″	38°07′58.62″	6.6	1.36	7.29	1.5	0.3	15.5	2858	466	75.5	2.32	1.97	0.18	156.1	14.48

### Next-generation sequencing

PCR was performed using 454 sequencing adaptor-linked primers flanking V4 region of the 18S rRNA gene: A-528F (5′-gcctccctcgcgccatcag-GCGGTAATTCCAGCTCCAA-3′) and B-1055R (5′-gccttgccagcccgctcag-ACGGCCATGCACCACCACCCAT-3′) (adaptor sequences shown in lowercase) [34] by GeneWiz, Inc. (Beijing, China). PCR mixtures (50 µl) were prepared in duplicate and each contained 2 µl of DNA template, 5 µl of 10× PCR buffer (50 mM KCl, 10 mM Tris-HCl and 1.5 mM MgCl_2_), 200 µM of dNTP, 0.2 µM of each primer and 2.5 U Taq polymerase (Promega, Madison, WI, USA). The PCR thermal regime consisted of an initial denaturation of 3 min at 94°C, followed by 30 cycles of 30 s at 94°C, 30 s at 60°C, 1 min at 72°C and a final cycle of 5 min at 72°C. PCR products were pooled and purified with the Qiaquick gel purification kit according to the manufacturer's instructions (Gene Company Limited, Beijing, China).

### Analysis of 454 pyrosequencing data and clustering of sequence reads into OTUs

The 454 sequencing reads were processed using the Qiime (Quantitative Insights into Microbial Ecology, v1.3.0) pipeline as flowgrams (.sff files). The data was processed following the steps recommended in the Qiime processing 18S data tutorial. In order to increase the quality of the raw sequences, reads shorter than 300 bp were excluded from the analysis to guarantee the analysis of the whole V4 region. Sequences longer than 670 bp (expected amplicon size) and those with more than one uncertain base (N) were further removed. The following parameters settings were used, namely an operational taxonomic unit (OTU) threshold of 0.97, 2 as maximum number of primer mismatches, 0 ambiguous bases, a maximum length of homopolymer run of 10, 300 nucleotides as a minimum sequence length and 670 as a maximum sequence length. The multiplexed reads were assigned to samples based on their nucleotide barcode. The data were then de-noised using the de-noiser wrapper within Qiime to remove the sequence errors characteristic of 454 sequencing machines. Chimeras were identified using ChimeraSlayer [50] and rejected from the dataset before construction of the OTU table. The OTUs were assigned using UCLUST [51], an open reference OTU picker within Qiime, a representative set of sequences was then generated and these sequences were assigned taxonomy using the SILVA release 115 database.

Another clustering process was to apply the NDIST module of AmpliconNoise [52] with default parameters to generate the distance matrix of all Needleman–Wunsch pairwise sequence alignments. Then, the average linkage option in the AmpliconNoise FCluster module was used to cluster reads into OTUs. In addition, we ran CD-HIT-OTU, a recent clustering algorithm for 18S rDNA pyrosequence data, with default parameters (including –e 0.0025) on the OTU finder web server (http://weizhong-lab.ucsd.edu/metagenomic-analysis/server/cd-hit-otu/).

### The Closest environmental match (CEM) and the Closest cultured match (CCM) available

We explored the 18S rRNA gene novelty of the dataset by BLAST identity search against GenBank sequences (search May 2014). The identity of each single sequence was related to both the closest environmental match (CEM), and the closest cultured match (CCM) available in GenBank. Histograms [15], [24], [53] were used to assess the degree of novelty comparing salinity gradient (Hypersaline lake vs. Mesosaline lake).

### Phylogenetic analysis

Sequence alignment of amplicon reads was performed with MegAlign implemented in DNAStar 6.0 software package (DNASTAR, Madison, USA) and then was confirmed visually by BioEdit 7.0.9 [54]. The ambiguous regions of alignment were discarded and eventually 733 nucleotide bases were obtained. Phylogenetic tree was reconstructed based on the full alignment of 733 sequences by using approximately Maximum Likelihood (ML) analysis in FastTree 2.1.3 [55]. For FastTree 2 analysis, a heuristics search strategy was employed with an estimated rate of evolution for each site (the “CAT” approximation), minimum-evolution subtree-pruning regrafting (SPRs), and maximum-likelihood nearest-neighbor interchanges (NNIs). Bootstrap ML analysis was carried out using 1000 pseudo-replicates. Trees were edited with the online tool iTOL [56].

### Other Statistics

For statistical analysis, the environmental parameters were transformed to avoid skewed data distributions: ion concentrations were arcsine transformed, other chemical parameters were log10 transformed; pH, altitude, and latitude were not transformed. We used principal component analysis (PCA) on chemical parameters (ion concentrations, pH, and conductivity) to display the main gradients in chemical parameters by CANOCO program (57). Significant marginal effects were analyzed by running separately a canonical correspondence analysis (CCA) on the OTU using square root transformation for each of the environmental factors separately (i.e. ion percentages, pH, altitude, TP, TN, TDS) by CANOCO program [57]. The data set generated in this study has been deposited at GenBank’s Short Read Archive (SRA) under Accession No. SRA178606.

## Results

### Characteristics of the studied lakes

**Figure 2:**
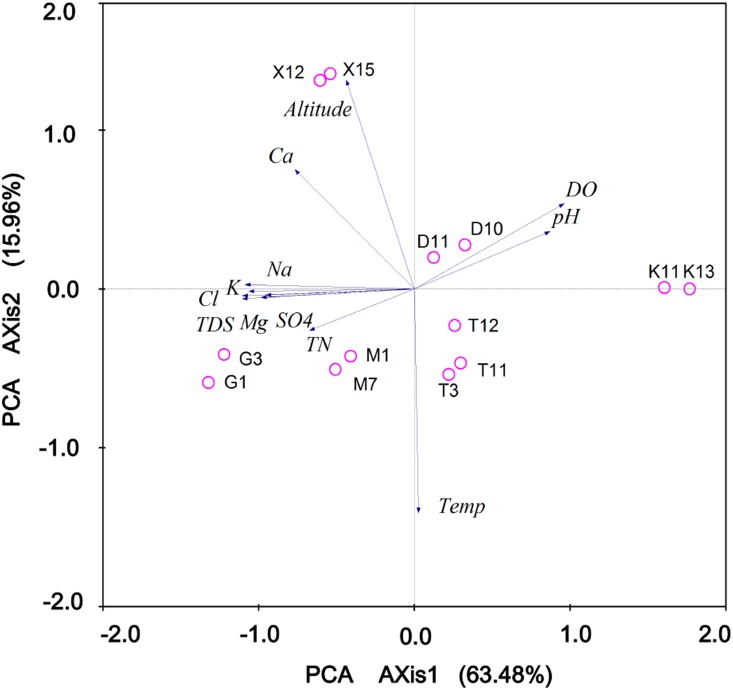
PCA analyses on DO, pH, TN and ion percentages of investigated Tibetan lakes. DO dissolved oxygen, TN total nitrogen, TDS total dissolved solid. K, Na, Ca, Mg, Cl, SO4 was represent for ions potassium (K^+^), sodium (Na^+^), calcium (Ca^2+^), magnesium(Mg^2+^), chloride (Cl^−^), and sulfate (SO_4_^2−^), respectively. The numbers 1 to 13 refer to investigated lakes, Lake Keluke (G1, G3), Lake Gahai (M1, M7), Lake Dasugan (D10, D11), Lake Tuosu (T3, T11, T12), and Lake Keluke (K11, K13), respectively.

The surveyed systems covered a wide range of variability in environmental conditions, including up to 40-fold differences in the salinity gradient (from 0 to approximately 46% salinity), altitude (from 2792 m to 3170 m), and in situ temperature (from 10.8 to 19.3°C) (Figure 1, Table 1). The Gahai, Gasikule, and Xiaochaidan lakes are more saline (hypersaline lakes >50 g/L) compared to the lakes Tuosu and Dasugan (mesosaline lakes, 20–50 g/L). The Lake Keluke is the only freshwater lake in this study. These six lakes are located in four different regions, with a minimum distance of 5 kilometers between them (Figure 1). Lake Gasikule is located in the northwestern part of the Qaidam Basin, where less precipitation and high evaporation have resulted in the highest salinity. Lake Dasugan, in which the ground water is considered the primary source of runoff, is located in the northern part of the Qaidam Basin. Lake Xiaochaidan is located in the northeastern part of the Qaidam Basin, whereas the other three lakes are located in close proximity to each other in the eastern part of the Qaidam Basin and have significantly different physical and chemical conditions (Table 1). Over the last century, Lake Xiaochaidan has been completely drained to exploit mineral resources and has been recovered by the reinjection of water. Lake Tuosu is connected to the freshwater lake Keluke by a short river; Lake Tuosu is expanding, resulting in altered salinities and phytoplankton communities. Table 1 lists the sampling sites, collected samples and physicochemical parameters that were measured. PCA of the lake environmental factors revealed that the first two components accounted for 79.44% of the total variation (PC1, 63.48%; PC2, 15.96%). There was a significant salinity gradient among the lakes, as observed by the high correlation between PCA axis 1 (Figure 2) and the chemical parameters (Table S1).

### Composition of the eukaryotic (nano- and picoeukaryotes) plankton community in saline lake

**Figure 3:**
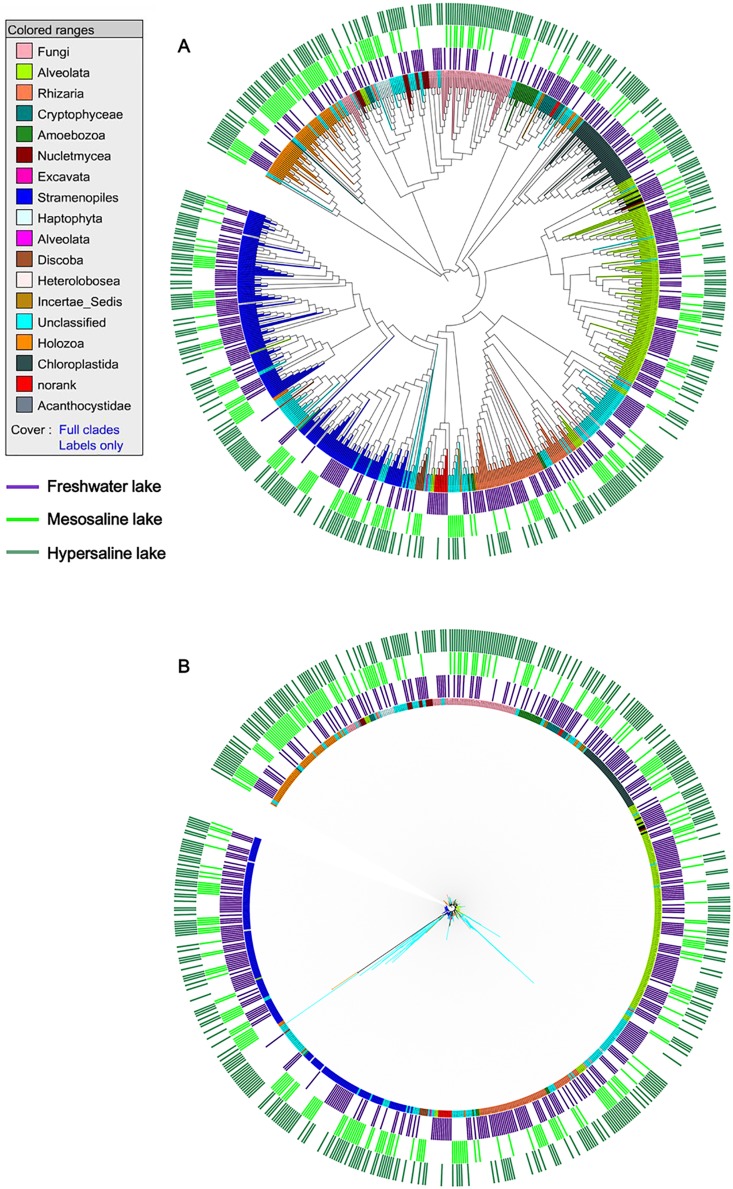
ML phylogenetic tree with 18S rDNA sequences of eukaryotes retrieved from the investigated lakes in the Qaidam Basin. The tree was constructed using 733 sequences. (A) Tree ignoring branch lengths and overlaid with colors with independent taxonomic assignations of sequences to an eukaryotic supergroup. The branches with cyan colors correspond to unclassified eukaryotes. (B) Same tree showing branch lengths, colored as before. The scale bar indicates 0.1 substitutions per position.

After quality filtering and preprocessing, 350 640 reads were obtained from the 13 sequenced samples in the study; approximately eighty-one percent, or a total of 286 360 reads, could be assigned to eukaryotic assemblages. Sequencing yielded highly variable results among the samples, ranging from 15 482 to 30 625 total reads per sample. A ML phylogenetic tree with all sequence reads provided a detailed picture of the diversity of Qaidam Basin lake eukaryotes (Figure 3A). To our surprise, at least 11.23% in total number of sequence reads could not be precisely assigned to any known eukaryotic taxonomic group. Further analysis showed that the 18S rRNA gene amplicon sequence were distributed among thirteen high-rank taxonomic groups and matched 70 eukaryal classes in freshwater lake (indigo in the outer ring) (Figure 3A, Table S2). Notably, at least 30 classes might be phylogenetically novel at least at the class level in freshwater lake. Most sequence reads from freshwater lake were affiliated with class Intramacronucleata (Alveolata), accounting for 29.50% of the total number of sequence reads. Eukaryotes from mesosaline lakes (lime in the outer ring) were distributed among eleven high-rank taxonomic groups and 38 eukaryal classes, whereas at least 16 classes could not be clearly attributed to known eukaryotic taxonomic groups (Figure 3A, Table S2). Craspedida (Holozoa) were the most prevalent class in mesosaline lakes, accounting for 26.52% of all sequence reads. The 18S rRNA gene sequences obtained from hypersaline lakes (seagreen in the outer ring) were distributed among eleven high-ranking taxonomic groups and matched 53 classes. The most prevalent class in hypersaline lakes was affiliated with Animalia (Holozoa), accounting for 21.98% of the total number of sequence reads. Notably, as was shown in Table S2, at least 35.85% of the total numbers of classes likely represented novel phylogenetic lineages at the class level.

**Figure 4:**
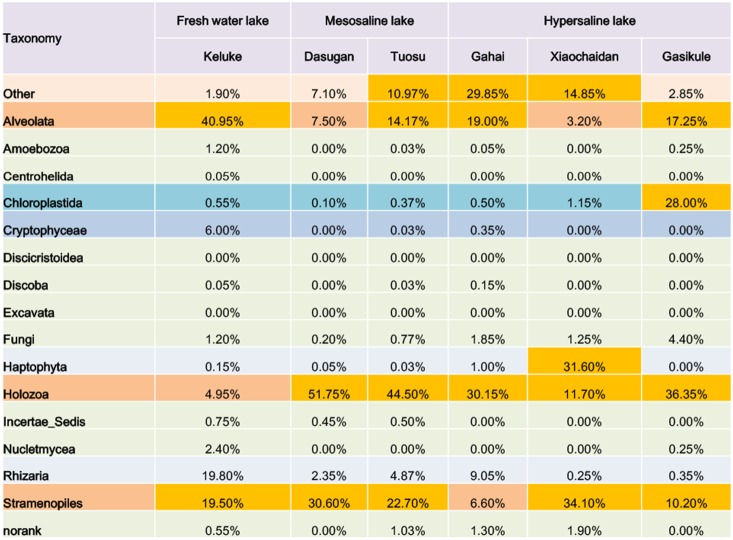
Distribution matrix with the proportion of sequence reads in investigated lakes with different physical and chemical conditions. Taxa were defined at Kingdom level. “Incertae sedis” is a term used for a taxonomic group where its broader relationships are unknown or undefined. “other” means group that could not be precisely assigned to any known eukaryotic taxonomic group. “norank” means group that could not be precisely assigned to eukaryotic taxonomic group at least at the kingdom level.

Holozoa were the most common kingdom in mesosaline Lake Tuosu, accounting for 44.50% of the total number of sequence reads (Figure 4). Stramenopiles were the second most prevalent kingdom, accounting for 24.2% of the total number of sequence reads. Approximately 10.97% of the sequence reads were new sequence types, defined as unclassified eukaryal clusters. The most prevalent kingdom in mesosaline Lake Dasugan was affiliated with Holozoa, accounting for 51.75% of the total number of sequence reads. Figure 4 showed that approximately 7.1% of the sequence reads were not classified into any of the known eukaryal clusters in Lake Dasugan.

There were approximately 14.85%, 2.85%, and 29.85% sequence reads defined as unclassified eukaryal clusters in the hypersaline lakes Xiaochaidan, Gasikule, and Gahai, respectively. Most sequences from Lake Xiaochaidan were affiliated with Stramenopiles (34.10%) (Figure 4). Haptophyta are the next most prevalent kingdom in Lake Xiaochaidan, accounting for 31.60% of all sequence reads. However, the most abundant sequence reads were mainly represented by Holozoa (36.35%), Chloroplastida (28.00%), Alveolata (17.25%), and Stramenopiles (10.20%) in Lake Gasikule. Holozoa is also the most common kingdom in Lake Gahai, accounting for 30.15% of the total number of sequence reads. The qualitative species composition of the abundant biosphere of Lake Gahai is shown in Figure 4. Alveolata were the third most prevalent kingdom, accounting for 19.00% of the total number of sequence reads.

**Figure 5:**
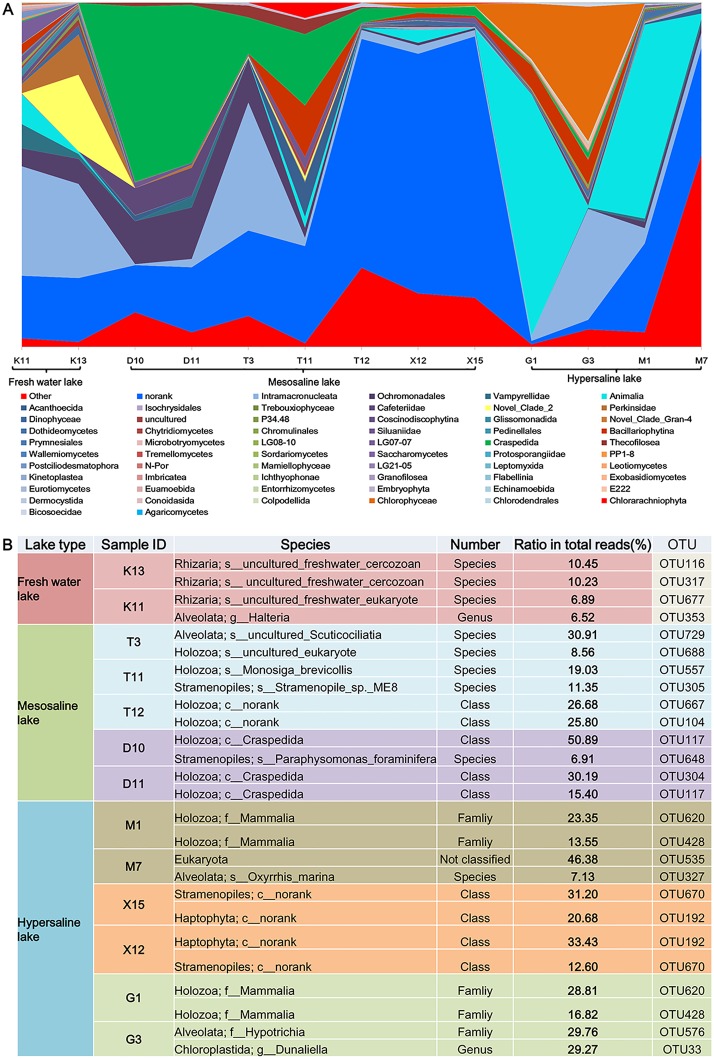
Taxa assignments at Class rank or below of investigated sample sits and the most abundant taxa in each habitat. (A) Taxa assignments at Class rank or below of investigated sample sits. (B) Taxa assignments at Class rank or below in each habitat. The class “other” means group that could not be precisely assigned to any known eukaryotic taxonomic group. The class “norank” means group that could not be precisely assigned to eukaryotic taxonomic group at least at the class level within kingdom. “Incertae sedis” is a term used for a taxonomic group where its broader relationships are unknown or undefined.

Taxonomic Composition of investigated lakes was shown in Table 2. Notably, the composition of the eukaryotic plankton community in different sampling sites of the same lake was significantly different (Figure 5, Table 2). Intramacronucleata (Alveolata) were the dominant class in sampling sites T3 (37.10%), whereas the most prevalent class in sampling sites T11 was Craspedida (Holozoa) (20.80%) (Table S2).

**Table 2:** Statistics of Taxonomic Composition of investigated lakes.

	Samples	Kingdom	Phylum	Class	Order	Family	Genus	OTU
**Fresh water lake**	K11	12	15	50	86	98	112	343
	K13	11	13	33	53	59	71	165
**Mesosaline lake**	T3	8	12	30	43	50	59	128
	T11	10	12	28	40	46	49	111
	T12	7	9	22	32	35	40	95
	D10	5	9	22	27	30	30	75
	D11	7	11	24	35	40	42	105
**Hypersaline lake**	M1	9	12	36	62	73	86	238
	M7	8	12	26	38	40	42	101
	X12	7	10	24	34	40	43	103
	X15	7	10	24	31	35	38	95
	G1	7	13	31	56	69	78	144
	G3	8	12	32	59	74	85	174

### Most abundant taxa in different sampling sites

As shown in Figure 5, an *uncultured_freshwater cercozoan* exhibited the highest proportion in K13, accounting for 10.45% of the total number of amplicon reads. However, the most common taxa in the K11 sampling point were *an uncultured_freshwater Eukaryote*, accounting for 6.89% of the total number of amplicon reads. The most common taxa in the D10 sampling point belong to the class Craspedida, accounting for 50.89% of the total number of amplicon reads (Figure 5). In contrasts, another species that affiliated with the class Craspedida was prevalent at sample site D11, accounting for 30.19% of the total number of amplicon reads. At stations T3 and T11, the eukaryal communities consisted mainly of uncultured_Scuticociliatia, and Monosiga brevicollis, respectively (Figure 5).

Lake Gahai is located quite far from Lake Gasikule. However, the compositions of the most common taxa in these lakes were quite similar. As shown in Figure 5, one species that affiliated with the Mammalia family was prevalent at sample site G1 and M1, accounting for 28.81% and 23.35% of the total number of amplicon reads, respectively. However, in another hypersaline lake (X15), the most abundant sequences were affiliated with a species affiliated with phylum Bicosoecida (31.20%), which was also the next most common taxa in X12 sample site (Figure 5). As was shown in Figure 5, the eukaryotic plankton assemblage was dominated by a species from the phylum Pavlovophyceae at the X12 sample site, accounting for 33.43% of the total number of amplicon reads.

Abundance tables of OTU were used to calculate Shannon’s diversity index and Simpson’s evenness. As indicated by the Observed species rarefaction curves, (Figure S1), the shape of the curves indicates a trend of diminishing chance of finding new phylotypes as sampling continues. The estimated number of observed species varied between 86.20 and 250.80 (Figure S1). The highest diversity index was observed in Lake Keluke (Observed Species = 250.80), and the lowest diversity index was in Lake Dasugan (Observed Species = 86.20). The Chao1 estimator was calculated to predict the total number of OTUs (richness) in the water samples. The taxonomic richness estimated for the six lakes was 267.50, 208.14, 199.21, 101.75, 117.80, and 119.35 for Lakes Keluke, Gasikule, Gahai, Dasugan, Tuosu, and Xiaochaidan, respectively (Figure S1). Considering the mean estimated richness in DNA datasets, Lake Keluke is the most diverse lake, closely followed by Lake Gsikule. Notably, eukaryotic plankton communities were more diverse in the hypersaline lakes compared to the mesosaline lakes.

### Cluster Analysis

Phylogenetic tree reconstruction using the maximum likelihood (ML) with all sequence reads elucidated the diversity of eukaryotic plankton assemblages in lakes located in the Qaidam Basin (Figure 3A). There were approximately 1.90%, 9.04% and 15.85% OTUs defined as unclassified eukaryal clusters in fresh water lake, mesosaline lakes and hypersaline lakes, respectively. This highly supported tree was pivotal to place very divergent sequences that could not be classified into any known eukaryal class that could not be identified by BLAST. Interestingly, some novel OTUs affiliated within a given taxonomic group. As shown in Figure 3A, about twelve novel OTUs were related to Holozoa groups. Nevertheless, at least four OTUs in mesosaline lake and eighteen OTUs in hypersaline lake could still not be related to any eukaryal taxonomic group, not even to a supergroup, and occupy highly unique branches in this phylogenetic analysis (Figure 3B). Phylogenetic analysis indicated that most OTUs from hypersaline, mesosaline, and freshwater lakes tended to form separate groups and shared rather low similarities (Figure 3).

### Novelty in each eukaryal class

**Figure 6:**
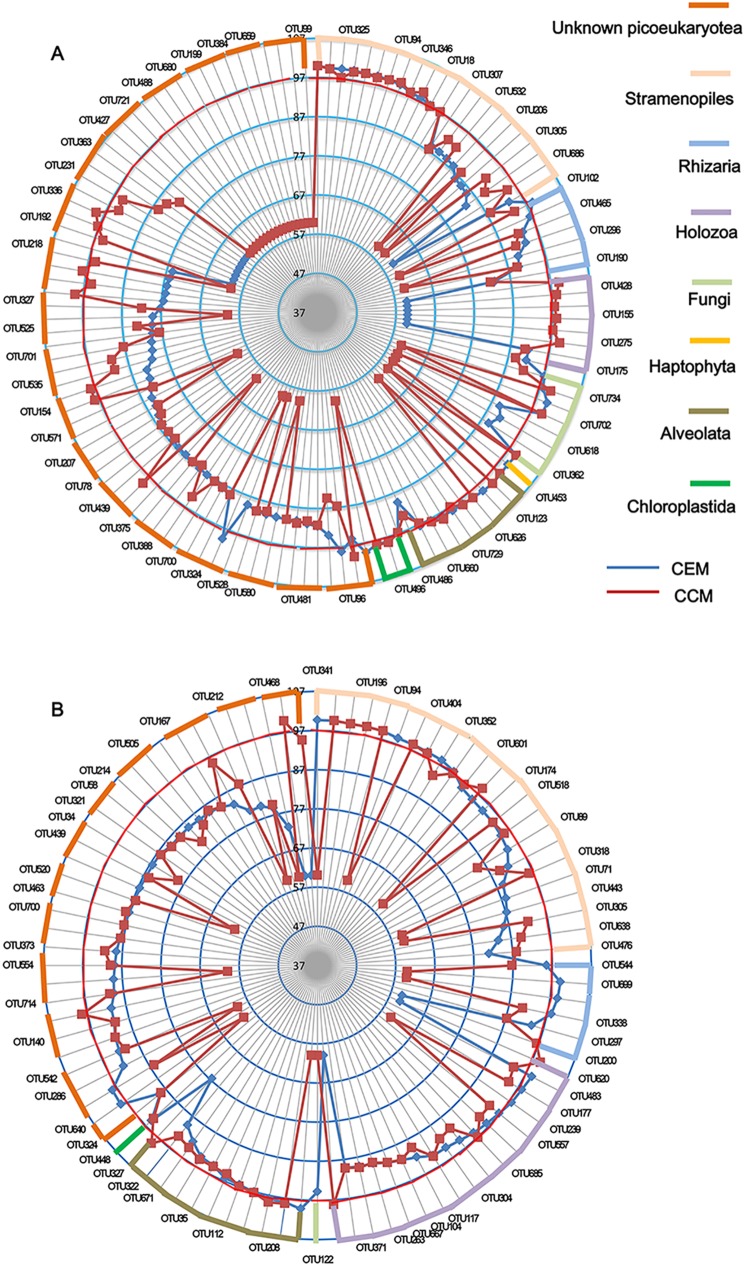
Novelty pattern plot for the different eukaryal classes found in mesosaline lake and hypersaline lake. (A) mesosaline saline, (B) hypersaline. Closest environmental match (CEM) and the closest cultured match (CCM) available in GenBank (BLAST search, May 2014). Dots below 61% similarity in both axes indicated no similarity sequences.

After water samples were obtained and sequenced, the identity of the different microorganisms was analyzed by BLAST search. For each OTUs, the similarity to the CEM and the CCM was recorded (Figure 6). The results were further classified according to salinity concentration (i.e., mesosaline vs. hypersaline, Figure 6A, B). The average CEM similarity (83%) was slightly lower than the average CCM similarity (83.87%) in hypersaline lakes. However, the average CEM similarity (89.96%) was much higher than the average CCM similarity (86.40%) in mesosaline lakes. We then analyzed the novelty of the taxonomic composition of the different assemblages. Each phylogenetic group exhibits a different novelty pattern, as observed with the supergroups Alveolates and Stramenopiles (Figure 6). In the kingdom Stramenopiles, the median genetic diversity values were ≥75% for both cultured and environmental matches, with similar sequence identity distributions in hypersaline lakes. Conversely, only approximately 35% of the Stramenopiles group in mesosaline lake matched both cultured and environmental sequences. As shown in Figure 6, dots below 80% similarity in both axes indicated highly divergent novel sequences. Notably, we observed that in mesosaline lakes (Figure 6A), at least four sequences had no similarity to CCM or CEM data, whereas in hypersaline lakes, up to eighteen OTUs yield generally low similarity values (Figure 6B). These OTUs were either not successfully cultured in the laboratory or have not been observed in nature.

### Distribution of OTUs and Influence of Physicochemical Parameters

Lake Keluke contained the highest number of OTUs unique to one lake, which account for approximately 59.92% of all OTUs (Figure S2). Venn diagrams comparing the number of common OTUs among samples showed that the hypersaline lake Gasikule and the freshwater Lake Keluke had a relatively large overlap (approximately 137 OTUs) compared to other lakes. The smallest proportions of OTUs were shared between the freshwater Lake Keluke and the mesosaline Lake Dasugan (approximately 47 OTUs). OTUs that were present in all conditions were rare: only six such OTUs were observed in our data set.

**Figure 7:**
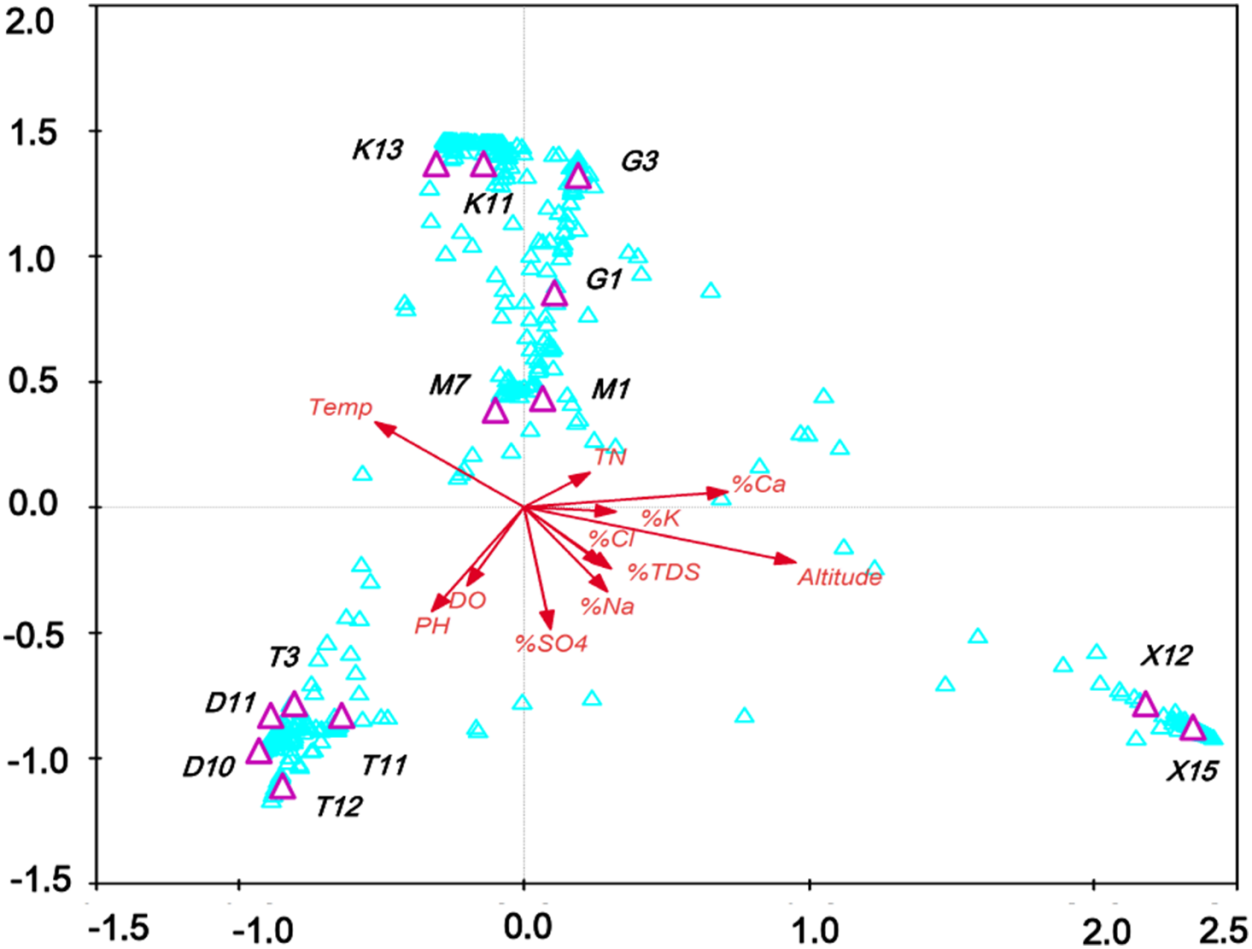
CCA biplots based on OTU and geographical or selected chemical parameters. Chemical parameters yield in a very similar spreading of sampling sites; DO dissolved oxygen, TN total nitrogen, TDS total dissolved solid. K, Na, Ca, Cl, SO4 was represent for ions potassium (K^+^), sodium (Na^+^), calcium (Ca^2+^), chloride (Cl^−^), and sulfate (SO_4_^2−^), respectively. In Figure, K, T, D, M, X and G referred to Lake Keluke, Lake Tuosu, Lake Dasugan, Lake Gahai, Lake Xiaochaidan and Lake Gsikule, respectively.

To analyze the influence of eukaryotic plankton community structure and other measured physicochemical parameters, a distinct CCA was generated from pooled habitat datasets. CCA analysis of chemical variables yielded four clusters, separated mainly by altitude and the percentage of calcium ions. The first cluster contained hypersaline lake sample points (X12 and X15); the second contained large hypersaline lake sample points (M1, M7, G1 and G3); the third cluster contained freshwater lake sample points (K11 and K13), and the fourth cluster contained mesosaline lake sample points (D10, D11, T3, T11 and T12). Altitude and the calcium-ion percentage affected the eukaryotic community structure in Lake Xiaochaidan, whereas the calcium ion percentage and TN were major factors in Lakes Gahai and Gasikule. pH and dissolved oxygen (DO) were the most important factors influencing the distribution of eukaryotic plankton assemblages in Lakes Dasugan and Tuosu (Figure 7).

## Discussion

The high-rank diversity observed in this study, both in terms of the eukaryotic supergroups detected and the presence and relative abundance of specific lineages, was typical of molecular surveys of eukaryotes [5], [7], [15], [25]. Shannon rarefaction curves reached plateau in the present study, indicates that more intensive sampling is likely to yield only few additional species. As not expected, the lake with the most extreme conditions (hypersaline lake Gasikule) had the highest OTU diversity, followed by the lake with the second highest salinity (Gahai). Although the actual numbers have to be regarded with caution, it seems clear that this study (and the many more to come with improved technologies) will significantly raise the higher limit of eukaryotes diversity.

### Taxonomic groups detected

Picoeukaryotes are probably the most abundant eukaryotes on earth. They are found in all lakes and oceans at densities from 10^2^ to 10^4^ cells/mL [58]–[61]. Alveolates, Fungi and Stramenopiles represent 65% of the total diversity and differ from the dominant groups known from microscopic studies in freshwaters Lake Pavin [62]. Recent research showed that most of the 18S rRNA gene sequences affiliate with Stramenopiles, Cryptophyta and Alveolata in several alpine oligotrophic lakes of the Central Pyrenees (Spain) [15], [63]. The taxonomic composition of the eukaryotic plankton assemblages, collected from freshwater Lake Keluke, differed from that reported in June 2012 from several alpine oligotrophic lakes of the Central Pyrenees (Spain) [15], where Stramenopiles, Cryptophyta and Alveolata dominated the 18S rRNA gene sequences. In the present study, Alveolata group represented a greater proportion of the community. Recent experiments by Mangot et al. (2013) showed that Almost 27% of the OTUs are affiliated with the Alveolates and more precisely the *Ciliophora* and *Perkinsozoa* taxa in freshwater Lake Geneva [64]. The dominance of Intramacronucleata within kingdom Alveolata in Lake Keluke highlights a difference in the composition of microbial eukaryotic communities between this lake and other lake.

From the general ecological principles it can be established that a more extreme environment is expected to be less species rich [65]. To our surprise, all the investigated saline lakes harbored remarkably diverse eukaryotic microbial communities, considering their higher salt concentrations. Mesosaline lake presented a different picture of the eukaryotic microorganisms community with freshwater lake. At least 7.10% of all sequences were related to unidentified eukaryal lineages in mesosaline lakes. Most 18S rRNA gene sequences obtained in mesosaline lakes were closely related to Holozoa (mainly Craspedida), differed from that reported in freshwater lake [58]–[61]. Genetic diversity of eukaryotic plankton assemblages has been well investigated in marine ecosystems. However, there is very few published studies utilised cloning-free high throughput sequencing to estimate the eukaryotic diversity of mesosaline lake. 454 pyrosequencing analysis reveal that Stramenopiles, Alveolata, Ciliates, and Prasinophytes were the dominant picoeukaryote groups in subtropical coastal waters [41]. The Stramenopiles constitute one of the major eukaryotic branches and include a vast number of heterotrophic and autotrophic groups with large ecological importance in the oceans [9]. Unlike in mesosaline lakes located in Qaidam Basin, in the Indian Ocean world, Alveolata and Stramenopiles are the most common groups, whereas Rhizaria are the next most abundant [24]. Recent experiments by Wolf et al. (2014) showed Alveolata (mainly Dinoflagellates) group are dominant in the sequence assemblage in the sub-Antarctic Zone [25]. These were not in agreement with the present result that Holozoa (mainly Craspedida) group was most prevalent in mesosaline lakes located in Qaidam Basin.

Hypersaline systems are overwhelming dominated by prokaryotes, with communities dominated by the haloarchaea [66]. However, eukaryotic organisms are also commonly present and might be play important ecological roles. In a study of 34 sites in different inland and coastal environments in Spain, most sequences are affiliated with Chloroplastida (mainly Chlorophyta), Alveolata, Stramenopiles, Opisthokonta, and Rhizaria, whereas Centroheliozoa, Haptophyceae, and Telonemida are less well represented [15]. Chlorophyta have adapted to specialised and extreme environments, such as deserts, arctic environments, hypersaline habitats [15], [67]. A study of high mountain saline lakes of the Eastern Tibet Plateau showed that most sequences are affiliated with Chloroplastida (mainly Chlorophyta) and Alveolata (mainly Dinophyceae) [7]. As we not expect, Chloroplastida group was only observed in high abundance in Lake Gasikule, whereas was less represented in other hypersaline lakes such as Gahai and Xiaochaidan. Our finding that Holozoa (mainly Animalia) were the most common kingdom in hypersaline lakes in the Qaidam Basin was contrast with a recent research that water samples were dominated (91%) by a novel cluster of the Alveolate in hypersaline Lake Tyrrell (Australia) [66]. These results were in agreement with previous reports that distinct biogeographical patterns are defined by the environmental conditions in particular regions [25]. The dominance of Holozoa (mainly Animalia) in water samples highlights a difference in the composition of eukaryotes between hypersaline lakes in the Qaidam Basin and other hypersaline environments.

It is of interest that Lake Xiaochaidan has the same salinity as Lake Gahai, but these lakes differed in the quantitative composition of their eukaryotic communities. Haptophyta were in high abundance in Lake Xiaochaidan, whereas present in lower numbers throughout coastal environments in Spain [15], Lake Gahai and Lake Gasikule. Several reasons might explain the different distributions of taxonomic groups in Lake Xiaochaidan compared to hypersaline lakes. First, Lake Xiaochaidan has been completely drained to exploit mineral resources in the last century, which has eliminated certain species, decreasing the abundance of the Holozoa group. Second, some species such as Alveolata, which are commonly observed in the freshwater Lake Keluke, might thrive because of freshwater injection. More interestingly, recent experiments by Charvet et al. (2014) showed that eukaryotic plankton assemblages in fresh waters were related to the Alveolata and in the lower saline waters were related to Stramenopiles [61]. However, further studies are required to confirm this hypothesis. Nevertheless, these data are consistent with previous conclusions that human activity is one of the greatest threats to these ecologically valuable habitats [3].

### Novelty analysis of environmental sequences

The novelty analysis revealed highly divergent sequences that appeared in the area of the dispersion plot with very low CCM and/or CEM values (Figure 6). A recent reports showed that 10% of the entire dataset showing <90% identity to any previously reported sequence in GenBank in 34 different coastal and inland saline ponds [15]. Notably, approximately 32% and 37.98% of the entire dataset in mesosaline lakes and hypersaline lakes, respectively, had <90% identity to previously reported sequences in GenBank (Figure 6). Most of these sequences formed long branches in the ML phylogenetic tree, although some could be confidently placed in a taxonomic group based on the dendrogram (Figure 3). The novelty level was also not equally distributed among the different taxa. In this study, Alveolata and Rhizaria contained the highest and lowest novelty, respectively, in hypersaline lakes. These results are not consistent with previous observations that Opisthokonta and Rhizaria contained the highest novelty and Chlorophyta and Alveolata contained the lowest novelty in 34 different coastal and inland saline ponds [15].

Furthermore, at least four OTUs in mesosaline lakes and eighteen OTUs in hypersaline lakes showed very low CCM and CEM scores, indicating that these OTUs are highly distantly related to any existing OTUs. As expected, phylogenetic analysis further showed that these OTUs occupy highly unique branches in phylogenetic tree (Figure 3B). Molecular approaches have been successfully used to estimate the diversity of eukaryotic plankton assemblages (largely, the protistan assemblages and picoeukaryotic communities) in marine ecosystems and have revealed a large and novel diversity of protists and other eukaryotic microorganisms [68]–[69]. Over a 4-year period, eukaryotic molecular surveys were performed and thousands of sequences were deposited [5], [7], [15], [25]. However, in this study, some OTUs still had no similarity to CCM or CEM data, yielding generally low similarity values. These OTUs could represent high-rank novel phylogenetic lineages and are obvious candidates for further research. Our data highlight incomplete culturing of the dominant eukaryotes in hypersaline environments in Qaidam Basin. From an evolutionary perspective, we are faced with very divergent sequences that could account for new, unexpected and fascinating evolutionary lineages [24].

### Influence of Salinity on Eukaryotic Plankton Assemblages

Understanding the spatial distribution of aquatic microbial diversity and the underlying mechanisms causing differences in community composition is a challenging task. Recent research suggested that distinct protist community assemblages are present in different large-scale water masses in the Southern Ocean [25]. Our data are consistent with this observation and have revealed the distinct distribution of eukaryotic plankton assemblages along a salinity gradient in the Qaidam Basin lakes. Notably, the eukaryotic plankton assemblage compositions were most variable between different sampling sites in the same lake (Figure 5). CCA analysis indicated that pH and dissolved oxygen (DO) were significantly correlated with the distribution of the eukaryotic plankton assemblages in Lakes Dasugan and Tuosu, whereas TN was a major factor influencing the taxonomic composition of eukaryotic plankton assemblages in Lakes Gahai and Gasikule (Figure S2). The present result confirmed that environmental factor strongly influences the taxonomic composition of eukaryotic plankton assemblages in inland waters [7], [60], [64].

The most important environmental gradients among the studied lakes were TDS in the present study. Previous reports also suggest that salinity and oxygen are important factors shaping the microbial composition in aquatic habitats [7], [56], [70]. Our results suggest that TDS defined distinct eukaryotic plankton assemblages among lakes, whereas other factors affected the distribution of eukaryotic plankton assemblages within lakes. This hypothesis consist with previous reports that chlorine and carbonate ion percentages were the most important chemical variables potentially structuring the diversity of eukaryotic plankton assemblages in Eastern Tibetan lakes [7]. Oxygen (presence or absence), pH, Na^+^, and K^+^ concentrations were significantly correlated with the OTU composition in Ethiopian soda lakes [27].

Inland saline lakes are extremely responsive to changes in climatic conditions and have significant ecological, economic, and cultural value; however, these lakes are threatened worldwide by diversion and pollution of their inflows, the introduction of exotic species, and economic development with changes in land use. This study shows that saline lakes in the Qaidam Basin, which are considered to be less species-rich most likely, harbored remarkably diverse eukaryotic microbial communities. Therefore, it is important to perform detailed studies and to develop active conservation strategies to preserve the microbial biodiversity in these areas. This result further highlights the major gap existing between the well-defined diversity and classification inferred from cultivated microorganisms, and their significantly larger natural diversity that is not yet well understood. These data provide the baseline information needed to further study the ecological context of the roles of microbial eukaryotes in the system.

## Supporting Information

Figure S1 **Estimating species richness of the investigated lakes.**
(TIF)

Figure S2 **Venn diagram showing the distribution of shared OTUs across lakes in the Qaidam Basin.**
(TIF)

Table S1 **shows Cumulative fit per species as fraction of variance of species.**
(DOCX)

Table S2 **shows Taxa assignments at class level of investigated lakes.**
(XLSX)
