# Supplementary figures and images for: High Genetic Diversity and Novelty in Eukaryotic Plankton Assemblages Inhabiting Saline Lakes in the Qaidam Basin
Source: PLoS One. 2014 Nov 17;9(11):e112812. doi: 10.1371/journal.pone.0112812 (PMC4234628; doi:10.1371/journal.pone.0112812)

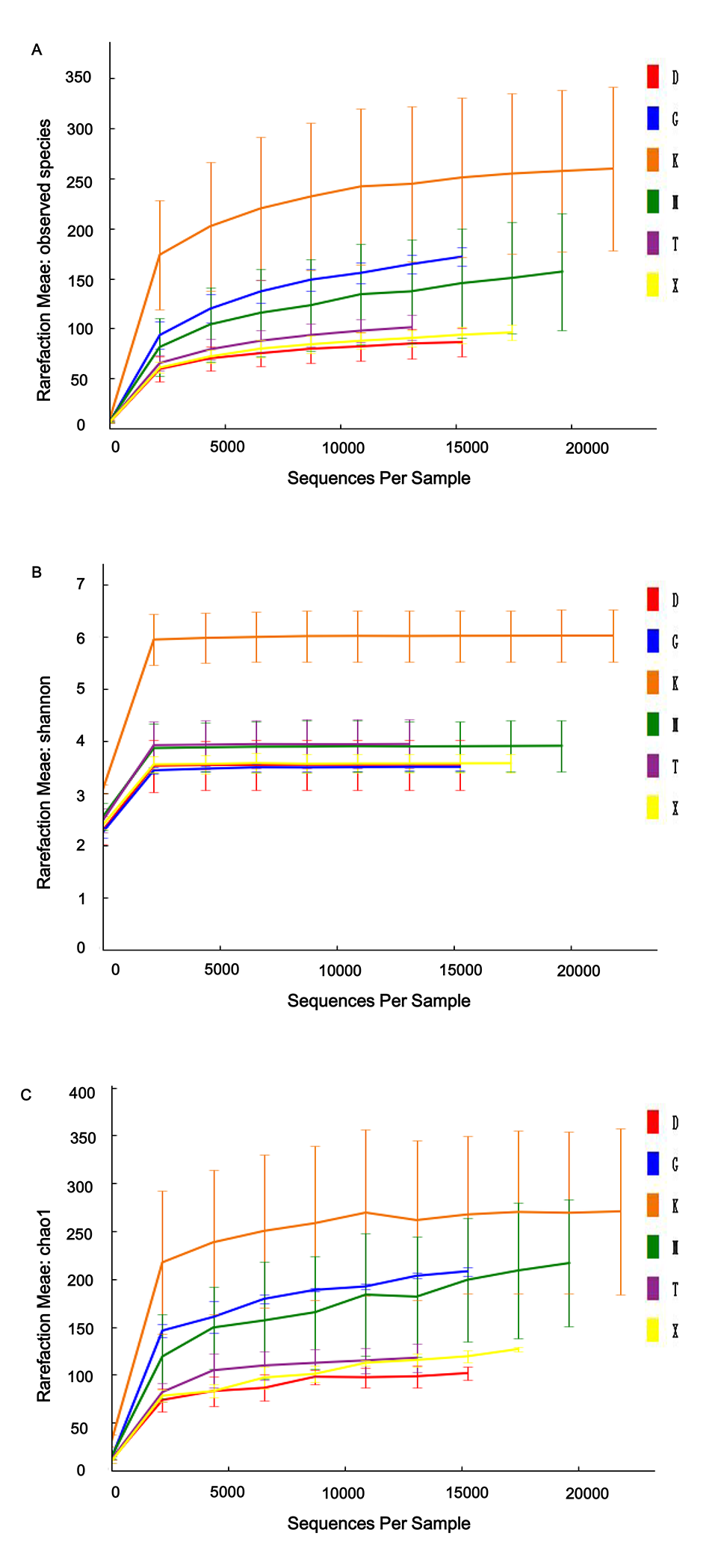

Supplement: Figure S1 — Estimating species richness of the investigated lakes. (TIF) [file pone.0112812.s001.tif]

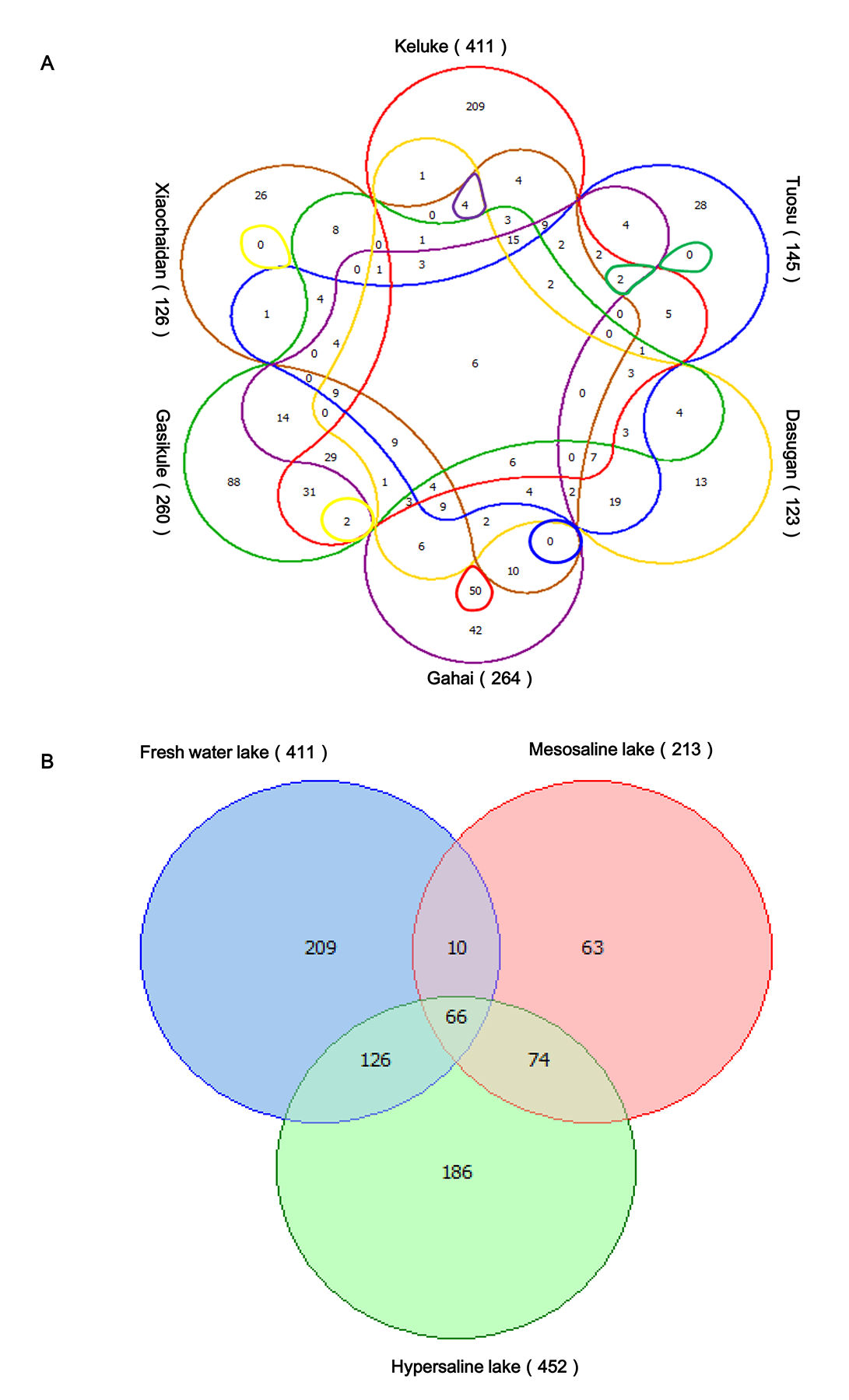

Supplement: Figure S2 — Venn diagram showing the distribution of shared OTUs across lakes in the Qaidam Basin. (TIF) [file pone.0112812.s002.tif]
