# Supplementary material for: High Genetic Diversity and Novelty in Eukaryotic Plankton Assemblages Inhabiting Saline Lakes in the Qaidam Basin
Source: PLoS One. 2014 Nov 17;9(11):e112812. doi: 10.1371/journal.pone.0112812 (PMC4234628; doi:10.1371/journal.pone.0112812)
Supplement: Table S1 — shows Cumulative fit per species as fraction of variance of species. (DOCX) [file pone.0112812.s003.docx]

Table S1 Cumulative fit per species as fraction of variance of species.

| Chemical parameter | PCA axis 1 score | PCA axis 2 score |
| --- | --- | --- |
| TDS | 0.9737 | 0.9752 |
| Cl^−^ | 0.9712 | 0.9719 |
| Na^+^ | 0.9420 | 0.9424 |
| K^+^ | 0.9005 | 0.9006 |
| Mg^2+^ | 0.7722 | 0.7733 |
| DO | 0.7402 | 0.8563 |
| SO_4_^2−^ | 0.7263 | 0.7268 |
| PH | 0.6071 | 0.6599 |
| Ca | 0.4696 | 0.6958 |
| TN | 0.3612 | 0.3884 |
| Altitude | 0.1526 | 0.8472 |
| Temp | 0.0007 | 0.7945 |

TDS: the total dissolved solid TN: total nitrogen TP: total phosphorus. K^+^, Na^+^, Ca^2+^, Mg^2+^, Cl^−^, SO_4_^2−^ was represent for ions potassium, sodium, calcium, magnesium, chloride, and sulfate, respectively.
